# Employment and sociodemographic characteristics: a study of increasing precarity in the health districts of Belo Horizonte, Brazil

**DOI:** 10.1186/1478-4491-7-56

**Published:** 2009-07-13

**Authors:** Maria Cristina Ramos de Vasconcellos Coelho, Ada Ávila Assunção, Soraya Almeida Belisário

**Affiliations:** 1 Municipal Health Secretariat of the City of Belo Horizonte, Brazil; 2 Faculty of Medicine of the Federal University of the State of Minas Gerais, Belo Horizonte, Brazil; 3 National Council for Scientific and Technology Development, Brasília, Brazil

## Abstract

**Background:**

The fundamental importance of human resources for the development of health care systems is recognized the world over. Health districts, which constitute the middle level of the municipal health care system in the city of Belo Horizonte, Brazil, deal with demands from all parts of the system. This research seeks to provide the essential features required in order to understand the phenomenon of increase in precarity of employment in these health districts.

**Methods:**

The legal and human resource management documents used by the Municipal Health Secretariat of the City of Belo Horizonte were adopted as the corpus for this research. In order to analyse the changes in employment (2002–2006), the data were collected from ArteRH, a computerized database dealing specifically with data related to human resources, which began operating in 2001. The workers were classified into permanent and non-permanent groups, and their contractual rights were described. Employment dynamics and changes were examined, concentrating on the incorporation of workers and on their social and employment rights during the period under study. The comparative data for the two groups obtained were presented in frequency distribution tables according to type of employment, sex, age group, level of education and wages from 2002 to 2006.

**Results:**

There was a clear difference between the permanent worker and non-permanent worker groups as regards existing guaranteed employment rights and social security. The increase in the number of non-permanent workers in the workforce, the growing proportion of older workers among the permanently employed and the real wage reductions during the period from 2002 to 2006 are indicative of the process of growing precarity of employment in the group studied.

**Conclusion:**

It is a plausible supposition that the demand for health reforms, along with the legal limits imposed on financial expenditure, gave rise to the new types of contract and the present employment situation in the health districts in Belo Horizonte.

## Background

The fundamental importance of human resources in enabling health systems to fulfil their aims is recognized the world over in studies and documents from a variety of institutions [1,2]. Employment and work protection constitute a fundamental policy to ensure better conditions for professional development in this sector, and they interact with the challenges of establishing a new model for the provision of health care [3].

The standard employment contract or typical job concerns the work carried out for one single employer. The typical job is based on an agreement made in an employment contract between employer and employee for work carried out in a specified place determined by the employer, for an indeterminate period, with specific tasks defined and carried out on a continuous, full-time basis according to the existing employment legislation [4]. A job without a standard employment contract can be considered to be precarious.

Precarious jobs are unstable, short-term, offer almost no possibility of promotion or a career, and have lower remuneration and fewer labour rights (holidays, wages, retirement benefits, etc.) in comparison with jobs where there is a standard employment contract [5-7].

In the world at present, the workforce is distributed unequally as regards the conditions of the employment contract, which are connected to different levels of conditions of work (hours of work, access to information about workplace hazards, rights during periods of sick leave, etc.). The negative effects of precarious work on safety at work and the quality of service provision are well-known [8,9].

In the case of public institutions, precarious jobs are those that have been put out to third-party contracts and are therefore subject only to operational control by managers. As a result, they do not have the legal rights that a contract guarantees for those who were selected by means of an open, competitive examination [10].

The present research seeks to examine the dynamics of employment and the changes in the health districts (HDs) of the Municipal Health Secretariat of the City of Belo Horizonte (MHS-BH), concentrating on an investigation into the incorporation of workers and the social and employment rights in place during the period under study. The HDs, which constitute the middle level of the municipal health system in Belo Horizonte, must meet the needs of the primary, secondary and tertiary levels of health care. The technical and management support required for carrying out these activities makes up part of the mission of the technical staff of the district management, either via its own professionals or temporary contractors.

This article was written with two aims in mind: (1) to determine the contractual rights of permanent workers and non-permanent workers in the HDs; (2) to examine the profile of the permanent workers and non-permanent workers in the HDs as regards the composition and distribution of the following variables: sex, age group, level of education, type of job and the purchasing power of the salaries earned by doctors, dentists and middle-level technicians. The results for each of the above-mentioned aims will be presented in separate sections.

## Methods

In order to examine the features of the contracts dealing with employment protection and the guarantees for specific social rights of workers as a whole, research was carried out making use of a set of documents dealing with relevant legal provisions and the management of human resources in the MHS-BH as its corpus.

As regards the study of the dynamics of the incorporation of workers, it was decided to describe the demographic characteristics of employment relating to the group consisting of 724 professionals employed by HDs.

### Selection of documents

The data concerning employment protection and guarantees of specific social rights of the workforce as a whole were obtained from the following documents: rough drafts of temporary contracts from the MHS-BH, drawn up in accordance with Municipal Laws 6.833/1995, 7.125/1996, 7.523/1998, 7.645/1999 e 9.011/2005; and the Statute of Public Service Workers of Belo Horizonte Trade Union, dealing with direct employment in management (Law no. 7169/1996). These contracts deal specifically with each occupational category in the HD of the MHS-BH and were analysed separately.

### Selection and source of the sampled

The period under analysis (2002–2006) coincides with the availability of data from ArteRH, a database dealing specifically with human resources, which began processing operations in 2001. ArteRH keeps an up-to-date register of human resources at MHS-BH that is not limited to any particular type of employment or length of the working day.

The tables had the following fields: Name, Employment Roll Number, Address, Telephone, Length of Working Day, Shift and Weekly Working Hours, as well as information about salaries. The data about the remuneration stipulated by contract were obtained from another source in the MHS.

During the period under analysis, the employment situation at the head offices of nine municipal health districts was examined, including managers and their teams, totalling 724 persons in 2006. The following occupations of the staff employed at that time were included in the investigation: doctors, dentists, high-level technical health staff (nurses, social workers, psychologists, pharmacists, veterinary surgeons, biologists, physiotherapists and occupational physiotherapists), health inspectors, assistant health staff (nursing assistants, laboratory assistants and dental assistants), management assistants, office assistants, doormen, watchmen/(security) security staff, typists, employees on work experience and health visitors not employed in their specific area and managers.

Health visitors, workers at the Sterilization Centre at one of the HDs and drivers were excluded because of the very different nature of their occupational duties, compared to those workers directly involved in the end mission of the HDs.

In order to evaluate the contractual remuneration, it was decided to select four formally recognized occupational categories on the staff of the Belo Horizonte Municipal Authority: doctors, dentists, high-level technical health staff and assistant health staff. The values analysed refer to the basic annual salary for each year.

The changes in contractual salaries and related purchasing power were analysed by means of an income deflator produced by institutions specialized in the study of employment in Brazil, which contained three correction factors: alteration of the reference date, by centring the index on the first day of the month; alteration of the value for July 1994 because of the change in the unit of currency that took place at that time; and expansion of the series to periods prior to its initial date [11].

The correction of nominal salaries is intended to deal with the salary from any point in time at constant prices and is justified by the differential changes in prices. This technique makes it possible to make comparisons between two moments in time in order to find out whether workers' purchasing power changed during that period.

It was not possible to study the existence and the extent of multiple employment, since the HR Management Module in the ArteRH database at the MHS-BH did not contain this information.

### Analysis of the data

Two main categories were used for the analysis of the data: full-time workers and part-time workers.

According to the Belo Horizonte Municipal Authority (BHMA) Public Service Worker's Regulations, a permanent worker is someone who is on the staff of the SUS-BH (National Health Service-Belo Horizonte) (municipal or municipalized) and was admitted to this service by public, competitive examination [12]. Permanent workers can be employed either full-time or part-time.

A non-permanent worker is someone who holds a political appointment (who can be freely hired and dismissed by whoever appointed him or her), someone who has a temporary contract, i.e. is subcontracted, is employed to provide a specific service or is a trainee. Non-permanent workers can be employed either full-time or part-time.

In order to produce Tables 1 and 2, which present the data regarding social employment protection, the types of contract were compared on the basis of social and employment rights. After reading all the documents comprising the corpus of the research, the aforementioned type of protection was selected for each of the categories of workers (permanent worker and non-permanent worker). The non-permanent workers were then classified according to the entity in which they were employed.

**Table 1:** Types of employment rights for the categories of permanent and non-permanent workers in the UHS-BH, 2002–2006

**Employment rights**	**Category of employment**				
	
	Non-permanent worker				Permanent worker
	Political appointment	Temporary contract	Subcontract	Trainee	Municipal Health Secretariat of the City of Belo Horizonte (SMSA-BH)

Entry	Nomination	Application	Application	Selection	Public competitive examination

Working week	40 hours exclusive contract.	40 hours per week	44 hours	20 hours (trainee) 30 hours (subcontractor)	20 hours or more

Holidays	25 working days	20 days every 12 months (when less than 3 absences during the period)	30 calendar days	Not specified	25 working days

13th Salary	1/12 year worked	1/12 year worked	1/12 year worked	Not specified	1/12 year worked

Sick leave	Time necessary for recuperation	Maximum of 2 days per month	Time necessary for recuperation	Not specified	Time necessary for recuperation

Validity of contract or competitive examination	Duration of political mandate	6 months, renewable 4 times	Indefinite	6 months to 2 years (trainee) Indefinite (subcontractor)	Permanent after 730 days worked.

Prior Notice	Not specified	15 calendar days	30 calendar days	Not specified	30 calendar days

Increase	According to Public Service increments	Not specified	Collective negotiations	Not specified	Collective negotiations

Source: Produced by the authors from data provided by GGTE/SMSA-BH – 2007.

**Table 2:** Leave and other specific rights: political appointees and full-time workers, UHS-BH, 2006

**Political appointees**	**Permanent workers**
Leave	Leave
Maternity	Maternity
Adoption	Adoption
Infant feeding	Infant feeding
Paternity	Paternity
Taking care of sick family member	Taking care of sick family member
Accident at work	Accident at work
	Taking care of spouse or partner
	Military service
	Election candidate
	Personal business
	Professional training

**Entitlement**	**Entitlement**

None	Retirement
	Good attendance bonus
	Five-year length of service bonus
	Special workday for students
	Shorter workday to take care of dependent with special needs
	Transport voucher
	Meal voucher
	Time allowance for decease/death of relatives; blood donation, jury, military or administrative service; marriage; force majeure; voter registration or military conscription process and designated off-duty periods – compensation for hours worked in special cases.

Source: Produced by the authors from data obtained from the Belo Horizonte Municipal Authority Internet Site – 2007.

The following variables were analysed: method of admission, length of working week, holiday entitlements and length of holidays, 13^th ^salary entitlement, medical leave allowance, validity of the contract or competitive examination, right to advance notice and annual salary increment.

The data allowing analysis of the dynamics of employment will be presented in frequency distribution tables according to employment rights, type of occupation, sex, age group, level of education, time of service and remuneration for the period 2002–2006.

## Results

### Social employment protection

Non-permanent workers in HDs can be admitted to public service in different ways: (1) by nomination in the case of political appointments; (2) by application in the case of temporary contracts offered by the UHS management; (3) by application in the case of subcontracts; (4) by application and interview for trainee contracts. Permanent workers are admitted to public service by public competitive examination.

The non-permanent health district workers have a 40-hour working week, except for those on subcontracts, whose working week is 44 hours long. Trainees and subcontractors work for 20 and 30 hours, respectively. For permanent workers, the workday varies according to the level of education required by their position: (1) those holding jobs that demand a university education have a 20-hour working week; (2) other workers have a 30-hour working week, which can be extended to 40 hours.

Table 1 compares the types of employment rights for the categories of permanent and non-permanent workers from 2002 to 2006. The non-permanent, subcontracted Health District workers are entitled to 30 calendar days' holiday per year, while those who hold political appointments have 25 calendar days per year. Employees with temporary contracts are entitled to 20 calendar days per year, providing that they have not been absent more than three times during that same period and their six-month contract has been renewed. The trainees and subcontractors have no holiday entitlement. Permanent Health District workers are entitled to 25 calendar days' holiday per year.

After completing 12 months of work, both the non-permanent and permanent Health District workers, except subcontractors and trainees, receive a 13^th ^salary equal to 1/12 of the yearly salary.

The permanent and non-permanent Health District workers are entitled to sick leave for the time necessary for recuperation, except for those temporary employees who are hired on temporary contracts, who are entitled to only a maximum of two days' leave per month. Subcontractors and trainees have no sick leave entitlement.

Temporary subcontracted Health District workers have contracts with unlimited validity, while political appointees' contracts are valid only for the duration of the political mandate. Temporary contracts are valid for six months, but, at the employer's discretion, can be renewed up to four times for periods of six months. Trainees have contracts that are valid for six months to two years (depending on level of education). Non-permanent workers' contracts can be revoked at any time at the employer's discretion. Permanent Health District workers' contracts become permanent after they have completed 730 days in their job.

Permanent Health District workers and temporary workers on subcontract are entitled to 30 days' prior notice of dismissal, while those on temporary contracts are entitled to 15 days. Health District workers who are political appointees, subcontractors and trainees are not entitled to prior notice of cancellation of their contract.

Permanent Health District workers and temporary workers on subcontract are entitled to salary increases via collective negotiation. Salary increases for political appointees depend on there being increases for public service workers, while workers on temporary contracts, subcontractors and trainees are not entitled to salary increases.

The specific types of leave and other entitlements for political appointees and permanent workers are shown in Table 2.

### The dynamics of employment

In the period under investigation, the total number of workers rose from 467 (2002) to 724 (2006), an overall increase of 55.03%

The total number of non-permanent workers in 2002 consisted of 72 workers; in 2006 this number increased to 292, a growth of 305% during this period. In the same period the total number of permanent workers grew by 9.36% in 2002, from 395 to 432. In 2004 there were 439 permanent workers, but in 2005 this dropped by 8.13%, to 406. However, in 2006 this number increased to 432, representing growth of 6.40% compared to 2005.

**Figure 1:**
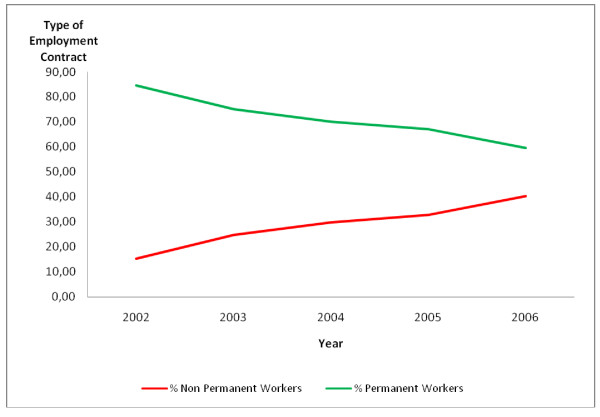
**Percentage distribution of workers in Belo Horizonte health districts according to type of employment contract, 2002–2006**. Source: Arte-RH – GPAR/GGTE/SMSA-BH – 2002–2006

It can be seen that during the years from 2002 to 2006 there was an increase in the percentage of non-permanent workers, which brought it close to the percentage of permanent workers (Fig. 1). Table 3 shows the ratio of permanent to non-permanent workers in the health districts in Belo Horizonte during the period 2002–2006.

**Table 3:** Ratio of full-time to part-time workers in Belo Horizonte health districts, 2002–2006

**Year**	**Ratio of permanent to non-permanent workers**
2002	5.49:1

2003	3.02:1

2004	2.35:1

2005	2.04:1

2006	1,48:1

Source: Arte-RH – GPAR/GGTE/SMSA-BH – 2002–2006

The ratio in 2002 was 5.49 permanent workers for each non-permanent worker (395 permanent workers to 72 non-permanent). In 2006, the ratio changed to 1.48 permanent workers for each non-permanent worker (432 permanent workers and 292 non-permanent).

Although there was a 55.03% overall increase in the total number of health district workers, the relationship between workers with non-permanent and permanent contracts changed over the period, indicating a tendency for greater growth in the former category compared to the latter.

**Figure 2:**
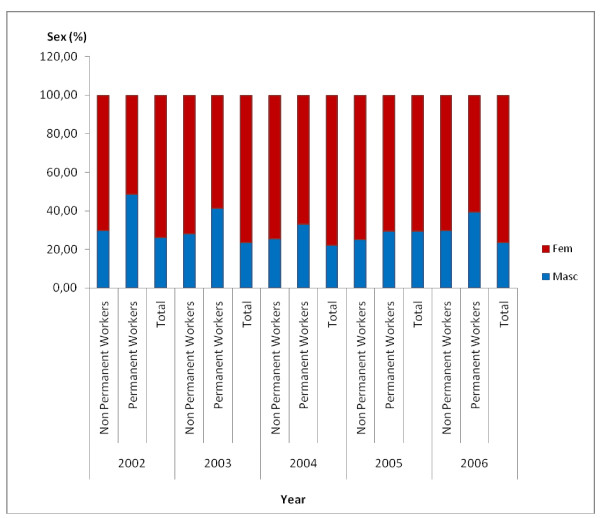
**Percentage distribution of workers in Belo Horizonte health districts according to sex, 2002–2006**. Source: Arte-RH – GPAR/GGTE/SMSA-BH – 2002–2006

In the case of distribution according to sex, there was a predominance of women in 2002 (70.24%). Fig. 2 shows the distribution of the total number of workers in the health districts according to sex from 2002 to 2006. The total number of both non-permanent and permanent women workers increased more than these categories of male workers during the period investigated. In 2002 there were 2.90 women for each man and in 2006 there were 3.24 women per man. A tendency for a reduction in the ratio between female and male non-permanent workers can be observed from 2005 onwards; this same tendency can be seen for permanent workers from 2004 onwards.

**Figure 3:**
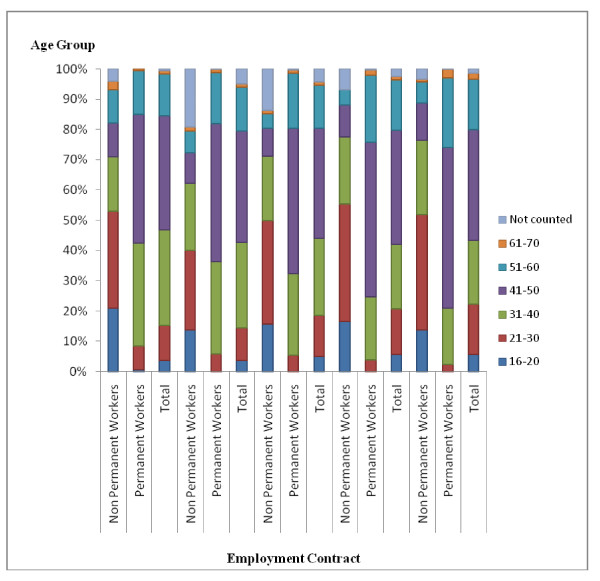
**Percentage distribution of workers in Belo Horizonte health districts according to age group, 2002–2006**. Source: Arte-RH – GPAR/GGTE/SMSA-BH – 2002–2006

Fig. 3 shows the distribution of the total number of workers according to age group for the period 2002–2006. During this period there was an increase in the number of non-permanent workers in all the age groups. In 2006, 79.07% of all the non-permanent workers were between the ages of 16 to 20, 21 to 30 and 31 to 40.

From 2002 to 2006, there was an increase in the number of permanent workers of all age groups. In 2006, 76.16% of the total number of permanent workers were between the ages of 41 to 50 and 51 to 60.

Over this period there was a decrease in the percentage of permanent workers in the 21 to 30 and 31 to 40 age groups and an increase of this category of workers in the 41 to 50 and 51 to 60 age groups. This demonstrated that permanent workers increasingly tended to come from the older age groups.

**Figure 4:**
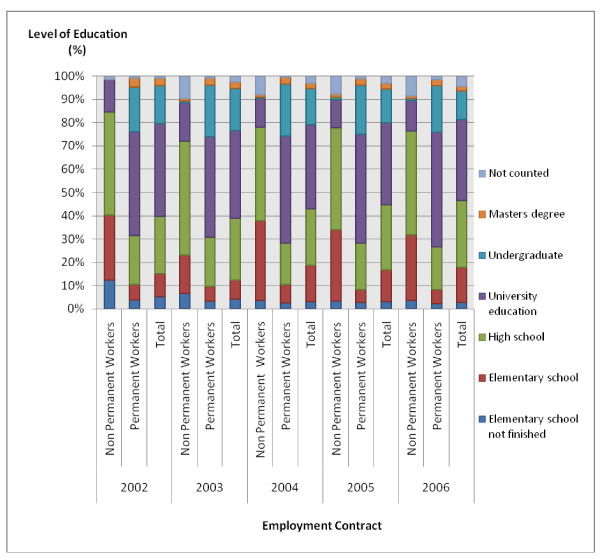
**Changes in the percentage of workers employed in Belo Horizonte health districts according to level of education, 2002–2006**. Source: Arte-RH – GPAR/GGTE/SMSA-BH – 2002–2006

In the case of both the permanent and non-permanent categories of workers it was found that, in the long term, there had been a decrease in the number of workers who had finished primary or secondary education and an increase in the number of workers who had completed university education (Fig. 4). However, this trend was found to be more in evidence in the case of permanent workers.

**Figure 5:**
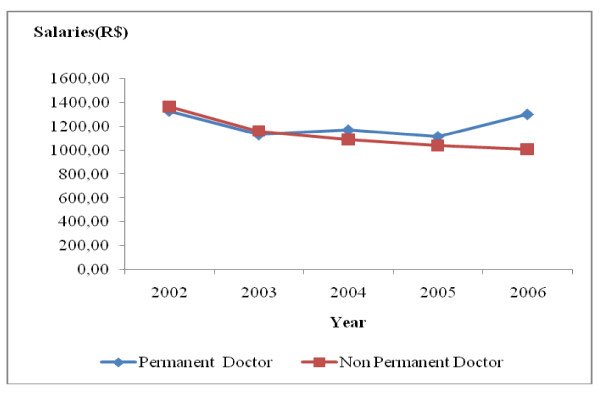
**Changes in doctors' salaries in Belo Horizonte health districts, 2002–2006**. Source: Arte-RH – GPAR/GGTE/SMSA-BH and deflator INPC (IPEADATA)

**Figure 6:**
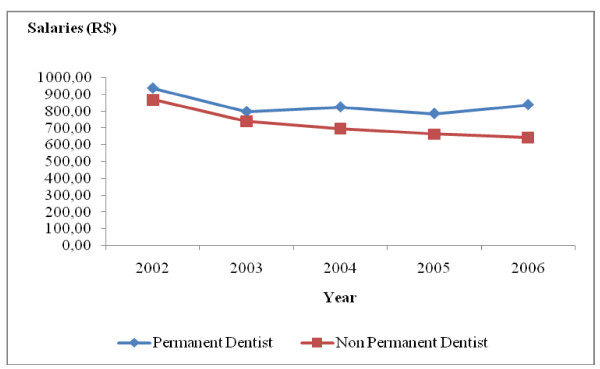
**Changes in dentists' salaries in Belo Horizonte health districts, 2002–2006**. Source: Arte-RH – GPAR/GGTE/SMSA-BH and deflator INPC (IPEADATA)

**Figure 7:**
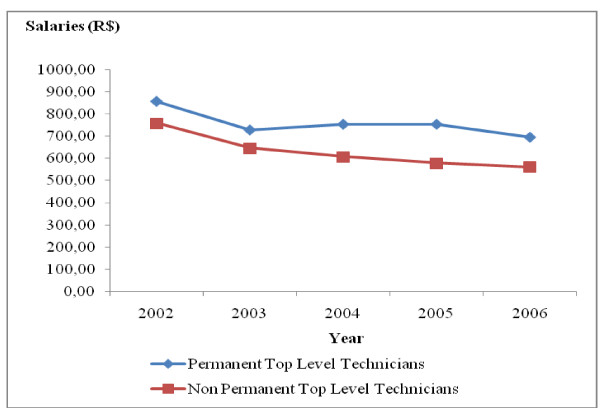
**Changes in top-level technicians' salaries in Belo Horizonte health districts, 2002–2006**. Source: Arte-RH – GPAR/GGTE/SMSA-BH e deflator INPC (IPEADATA)

**Figure 8:**
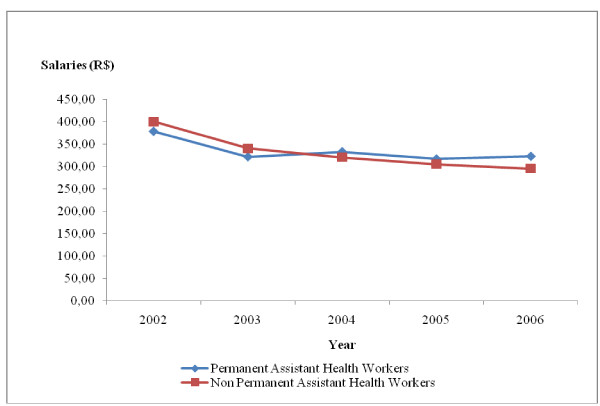
**Changes in assistant health worker's salaries in Belo Horizonte health districts, 2002–2006**. Source: Arte-RH – GPAR/GGTE/SMSA-BH and deflator INPC (IPEADATA)

During the period under study, there was a drop in the real salaries of all the occupational categories studied, especially in the case of non-permanent workers. For permanent workers, there was an increase in real salaries of all the occupational groups in 2004 and 2006, but without returning to the values obtaining in 2002. The real salaries of all the occupational categories of non-permanent workers dropped by 26.09% between 2002 and 2006. As regards those in permanent employment between 2002 and 2006, the real salary varied according to occupational category. It was 2.05% for doctors (Fig. 5), 10.54% for dentists (Fig. 6), 18.70% for high-level technical health staff (Fig. 7) and 14.61% for assistant health staff (Fig. 8).

## Discussion

The reform of the Brazilian health system increased the public health liabilities of municipal authorities and made it necessary to enlarge the workforce in order to implement the new health policies. Since the Municipality of Belo Horizonte was obliged to comply with the new requirements of the Family Health Programme but faced legal spending limits, it opted to contract for workers by means of alternatives to the standard employment contract. It is therefore reasonable to suppose that this situation explains why the increase in the number of workers was concentrated in the non-permanent category.

There was a rise in the total number of non-permanent workers, whose contractual rights and employment protection were reduced. These results are consistent with data from the Ministry of Health that show that approximately 800 000 health workers are in precarious employment, or almost 40% of the workforce in this sector [13]. These data led to Brito's [14] investigation into the three parts of the public health reforms that had still not been properly dealt with by management: the employment contract, work processes and work relations.

In Brazil, about 42% of the urban workforce does not have a valid standard employment contract [15]. In the health sector, which is no different from the general situation, there has been a rise in the incidence of unregistered wage employment at the social security agencies [16]. In addition to this, an increase in multiple employment has been the solution adopted to deal with the low salaries of this occupational category [17]. Stress and exhaustion are to be expected as the results of this situation [5].

Despite the negative features mentioned, the lack of structural unemployment in the health care sector in Brazil stands out. Between 1995 and 2000, there was an increase of 113 351 posts in this sector, representing a net growth of 13.9%. In 2000, 3.5% of jobs in the formal labour market (930 189 posts) were to be found in the health sector. There was a 50% net rate of growth in employment in the municipal health sector from 1995 to 2000 [18-20].

Recently there have been studies of the creation of new jobs brought about by the increased coverage of health services. The strong potential for formalizing employment in this sector is also clear when the level of non-permanent or short-term contractual employment is compared to the average for the Brazilian labour market. In spite of this, an analysis of the database from the research carried out by Dedecca et al. [21] showed that in 2000, 23 862 out of a total of 198 153 doctors had jobs that were not officially registered; the remainder were distributed among the categories of employers or self-employed and trainees. The distribution of occupations among the total of uncertified nursing assistants was as follows: 132 080 with official registration; 41 740 employed by the State; 32 305 not officially registered; 7230 on work experience and 766 in unpaid posts.

The predominance of women in both the full-time and temporary categories during the period studied matches both domestic and international tendencies. At present in the United States, women constitute 80% of the workforce in the health sector [22]. In the European Union, the percentage of women is around 77% [23].

In 2000, women occupied 73% of the health service jobs in Brazil [18]. In the metropolitan regions of six Brazilian cities (Belo Horizonte, the Federal District of Brasília, Porto Alegre, Recife, Salvador and São Paulo), the profile of health service workers is similar to this study in that female workers predominate, they are from age groups above 25 years and have a high level of education, which is frequently at university level. In 2004, health service workers in Belo Horizonte, the city that is the focus of this study, represented 5.5% of the economically active population, made up principally (73.7%) of women [24].

For Hirata and Préteceille [25] the growing acceptance of non-permanent work and temporary work is one of the possible causes of the greater presence of women in the labour market, because this makes it possible to reconcile domestic and paid activities, as well as allowing the employer greater flexibility as regards salaries.

In a study of the feminization of the labour market in general, Lavinas [26] questions whether this phenomenon is the result of changes in the nature of the female labour force or whether it derives from changes in the dynamics of the labour market. According to this line of reasoning, the present trend towards greater flexibility of salaries and the length of the working day favours women's insertion into the labour market, but therefore also reflects the increasing precarity of employment in Brazil. It would not be an exaggeration to state that, in Brazil, the existence of a higher level of education among women in comparison to men might be one of the possible explanatory factors for the feminization of employment in the health care sector.

As regards age group, the tendency for the youngest and oldest groups of permanent workers in the health districts to increase may result in lack of competence in responding to job requirements. In general, workers located at the opposite ends of the age pyramid can be considered to be less experienced – in the case of the youngest – or the weakest physically – in the case of the oldest – who are also less able to adapt, for example, to demands for versatility. If both situations are not adequately dealt with by human resources management, this may explain such undesirable consequences as loss of quality in the provision of services, greater exposure to areas of insecurity and higher levels of stress [27].

According to Girardi and Carvalho [18], the average age of health service workers in Brazil in 2000 was 38. The authors report that all the occupational categories in health work show signs of ageing in comparison with previous years. The same is true in the north-eastern State of Rio Grande do Norte in 2005 [28].

The fact that non-permanent workers were concentrated in the 16 to 20 and 31 to 40 age groups might be associated with young people's greater willingness or ability to comply with institutional norms and accept lower salaries [29].

Once again, the data in these findings are consistent with the national tendencies for the majority of employed workers to be in the 18 to 24 and 25 to 39 age groups. It is reasonable to attribute the rise in the rate of participation of the 60 year-old age group to postponement of retirement or to retired workers returning to the labour market as a strategy to replace the loss in family income resulting from the unemployment of one of its members [30]. The normal age of retirement in Brazil is 65 to 70.

As regards level of education, a tendency was found for an improvement in workers' qualifications regardless of the type of contract. The fact that non-permanent workers are concentrated among those who have finished secondary education can be explained by the presence of trainees carrying out management functions in the health districts. Bezerra reports that in the State of Rio Grande do Norte, more than 60% of the health sector was made up of workers who had finished secondary education and who were engaged in management activities or were working as health assistants [28].

A tendency for there to be an improvement in permanent workers' qualifications was observed. From 2002 to 2006, there was a reduction in the total number of permanent workers who had finished their secondary education and, in the same period, an increase in the total number of workers with university-level education. These data are similar to the data from the "Research into Employment and Unemployment in Belo Horizonte". According to this research, there was a rise in the number of employed workers who had obtained a primary education and an expansion of the number of workers who have a secondary education, thus representing a tendency for an improvement in employed workers' qualifications [30].

During the period, the real salaries of all occupational categories in the health districts fell, especially those of non-permanent workers. In 2004 and 2006, all the occupational categories in permanent work improved their real salary, but without regaining the level of 2002. Although the average salaries in the public services were higher than those in the private sector, there was a real drop in salaries in both sectors during the 1990s, thus making it clear that the labour market in Brazil had become more precarious [31].

## Conclusion

This research describes the changes in employment and sociodemographic characteristics of the workers in the health districts of the MHS-BH during the period from 2002 to 2006. The difference between full-time and non-permanent workers as regards employment rights and social protection is clear, thus confirming the tendency towards precarity in the case of part-time employment.

The rise in temporary employment, the tendency for the ageing of permanent workers and the drop in workers' purchasing power during the period from 2002 to 2006 are indicative of the process of increasing precarity of work and justify government policies aimed at reversing this tendency. Federal management has highlighted the damage caused by temporary employment contracts to the smooth operation of the health service [13]. Staff with stable jobs, recognized technical leadership, professional dedication and production of knowledge are the attributes of institutions that have acquired the ability to provide health care of good quality [32].

In general, non-permanent workers have a lower level of job security, less control over their hours of work, worse career prospects and limited access to training and education [5].

In Brazil from 1991 onwards, the state apparatus significantly degraded working conditions by increasing the possibility of more flexible employment contracts, profit sharing, flexible working days (Hour Banks), Sunday work and cuts in jobs and salaries.

Maintaining these work practices led to changes in public regulation of work contracts; on this new basis, part-time or fixed-term contracts, reduction in the social security contributions paid by small companies and public youth employment subsidies were permitted. Along the same lines, governments reduced investment in the inspection of work contracts, thus reducing the possibilities of companies being punished for not complying with the law [33].

As mentioned earlier, the National Health Service did not escape this situation. The increasing precarity of work in health care has been a source of concern for managers at all levels of government and has been a priority agenda item for the National Council of Municipal Health Secretaries [*Conselho Nacional de Secretários Municipais de Saúde *(CONASEMS) [34].

In order to reverse this situation, the National Programme to Reduce Precarity of Work (Programa Nacional de Desprecarização do Trabalho) was set up in the National Health Service in order to encourage stable work relations, guarantee workers' rights and to gradually eradicate the precarious work relations in this sector. This program produced publicity materials, helped to set up local committees, sponsored debates, established a support network, carried out an investigation of workers in precarious work situations and encouraged research on the impact of the Law on Financial Liability (*Lei de Responsabilidade Fiscal*) on reducing precarity of work in the National Health Service [35].

In the case of Belo Horizonte, various measures were adopted in order to minimize the situation regarding precarity of work and employment in the National Health Service-Belo Horizonte. The following items may be mentioned: (1) creation of the Health Education Centre (*Centro de Educação em Saúde*) in 2005 in order to provide training courses; (2) teleconferences and other types of educational methods to make it possible for specialist professionals to assist those professionals working in the basic health service to provide treatment for clinical cases; 3) presentation and discussion of the technical topics most relevant for daily work activities in the health units.

In addition to this, between 2002 and 2006, three public employment admissions exams were organized to select personnel for positions in the health service. It is also intended to organize another of these public admission examinations for doctors. All non-permanent contracts for career health professionals are being replaced by permanent contracts. To this we can add the law approved in 2007, which awarded salary increases for all the positions included in the Belo Horizonte Municipal Authority Health Jobs and Career Plan [36].

In the case of training and development of skills, additional problems are posed for human resources management. Lifelong learning is fundamental to professional performance and for being able to deal with the effects of changes in the workplace. Workers who do not have standard employment contracts may be carrying out functions that fail to make full use of their skills and capacities [4]. This situation as a whole can cause occupational stress [5].

These results may serve to guide human resources management in the sector, considering that the health districts are essential for the performance and development of the Unified Health Service in Belo Horizonte. Moreover, the activity of the workers in this area is of fundamental importance in bringing about a transformation of health practices and the quality of service provided to the public [37,38].

The variety of political, ideological and technical roles played by workers in the health districts would be strengthened in an environment where there was greater employment and work protection. In this regard, qualitative studies would be able to elucidate the effects of the situation described above and identify the levels of dissatisfaction found in the sector.

An understanding of the employment profile in the health districts is the first step towards development of a human resource policy directed at middle management. It opens up the possibility of new research into various aspects that were not dealt with in this study, such as ensuring that staff members have the correct aptitudes and training for the demands facing middle management, assessment of overtime and resizing of (full-time) staff.

## Competing interests

The authors declare that they have no competing interests.

## Authors' contributions

MC, AA and SB jointly formulated the study design. MC obtained the data. MC and AA were involved in the conceptualization, initial drafts and final write-up of the paper. All authors had access to all data in the study and had final responsibility for the decision to submit this manuscript for publication.

## References

[B1] Fritzen AS (2007). Strategic management of the health workforce in developing countries: What have we learned?. Human Resources for Health.

[B2] Alliance Mondiale pour les Personnels de Sante. Organisation Mondiale de la Santé (2008). Directives: mesures incitatives pour les professionnels de la santé. http://www.who.int/workforcealliance/knowledge/publications/alliance/Incentives_Guidelines%20FR%20low.pdf.

[B3] Brito PE, Padilla M, RígolI F (2002). Planificación de recursos humanos y reformas del sector salud. Educação Médica Superior.

[B4] Galeazzi IMS, Bastos RLA (2007). O trabalho por conta própria num contexto de precarização laboral. Dimensões da precarização do mercado de trabalho na Região Metropolitana de Porto Alegre.

[B5] New forms of contractual relationships and the implications for occupational safety and health. http://osha.europa.eu/publications/reports/206.

[B6] Hirata H (2000). Divisão sexual do trabalho: novas tendências e problemas atuais. Gênero no mundo do trabalho.

[B7] Ramos L, Reis JGA (1997). Emprego no Brasil nos anos 90.

[B8] Dussault G, Rigoli F (2002). Dimensiones laborales de las reformas sectoriales en salud. Sus relaciones con eficiencia, equidad y calidad. Revista Latinoamericana de Estudios del Trabajo.

[B9] (2000). The changing world of work. Magazine of the European Agency for Safety and Health at Work.

[B10] Nogueira RP (2006). Problemas de gestão e regulação do trabalho no SUS. Serviço Social e Sociedade.

[B11] Uma sugestão de deflatores para rendas obtidas a partir de algumas pesquisas domiciliares do IBGE. http://www.ipea.gov.br/pub/td/2002/td_0897.pdf.

[B12] Prefeitura Municipal de Belo Horizonte (1996). Lei 7169, de 30 de agosto de 1996.

[B13] Ministério da Saúde do Brasil (2006). Para subsidiar a discussão sobre a desprecarização do trabalho no SUS. Cadernos RH Saúde.

[B14] Brito P (2000). Impacto de las reformas del sector de la salud sobre los recursos humanos y la gestión laboral. Revista Panamericana Salud Publica.

[B15] Ramos L, Ferreira V (2006). Padrões espacial e setorial da evolução da informalidade no período 1991–2005. Pesquisa e Planejamento Econômico.

[B16] Cacciamali MC (2000). Globalização e processo de informalidade. Economia e Sociedade.

[B17] Medeiros SM, Rocha SMM (2004). Considerações sobre a terceira revolução industrial e a força de trabalho em saúde em Natal. Ciência & Saúde Coletiva.

[B18] Configurações do mercado de trabalho dos assalariados em saúde no Brasil. http://www.opas.org.br.

[B19] Machado MH, Lima NT (2005). Trabalhadores de Saúde e sua trajetória na Reforma Sanitária. Saúde e Democracia: história e perspectivas do SUS.

[B20] Cordeiro H (2001). Descentralização, universalidade e equidade nas reformas da saúde. Revista Ciência & Saúde Coletiva.

[B21] Dedecca CS, Rosandiski CS, Carvalho EN, Barbieri CV (2005). A dimensão ocupacional do setor de atendimento à saúde no Brasil. Trabalho, Educação e Saúde.

[B22] Centers for Disease Control and Prevention (2008). National Institute for Occupational Safety and Health. Health Care Workers.

[B23] European Agency for Safety and Health at Work (2003). Safety and Health Good Practice on-line for the Healthcare Sector.

[B24] O trabalhador da saúde em seis regiões metropolitanas brasileiras. http://www.dieese.org.br/notatecnica/notatec33saude.pdf.

[B25] Hirata H, Préteceille E (2002). La prise en compte de l'insécurité socio-économique dans les grandes enquêtes statistiques en France. BUREAU INTERNATIONAL du TRAVAIL Exclusion, Précarité, Insécurité Socio-Économique (Apports et débats des sciences sociales en France).

[B26] Lavinas L (2002). Perspectivas do emprego no Brasil: inflexões de gênero e diferenciais femininos. Emprego feminino no Brasil: mudanças institucionais e novas inserções no mercado de trabalho.

[B27] Froneberg B (2006). National and international response to occupational hazards in the healthcare sector. Annals of the New York Academy of Sciences.

[B28] Bezerra O (2006). Dinâmica e características do mercado de trabalho do setor saúde em Natal – RN.

[B29] Rodrigues FS (2006). O segmento de meia idade: análise sobre os desdobramentos do padrão de acumulação flexível em sua inserção produtiva. Bahia Análise & Dados.

[B30] Oliveira AM, Januzzi JM, Soares M (2006). Dez anos da Pesquisa de Emprego e Desemprego (PED) na região metropolitana de Belo Horizonte (RMBH): tempos difíceis para os ocupados. As várias faces do mercado de trabalho no Brasil.

[B31] Borges AMC (2004). Reforma do Estado, emprego público e a precarização do mercado de trabalho. Caderno CRH.

[B32] O'Dwyer GC (2002). Trabalho, ética e necessidades sociais em saúde. Política de recursos humanos em saúde.

[B33] Dedecca CS (2006). Flexibilidade e regulação de um mercado de trabalho precário: a experiência brasileira. Colóquio Internacional – Novas Formas do Trabalho e do Desemprego: Brasil, Japão e França numa perspectiva comparada.

[B34] CONASEMS (2006). Gestão do trabalho e educação na saúde. Teses e Plano de Ação.

[B35] (2007). Orientações gerais para elaboração de editais – processo seletivo público: agentes comunitários de saúde e agentes de combate às endemias.

[B36] Prefeitura Municipal de Belo Horizonte (2007). Edital 02/2007. Diário Oficial do Município.

[B37] Roncalli AG, Pereira AC (2003). O desenvolvimento das políticas públicas de saúde no Brasil e a construção do Sistema Único de Saúde. Odontologia em saúde coletiva: planejando ações e promovendo saúde.

[B38] Malik AM (1998). Gestão de recursos humanos.

